# Comparison of Common Postoperative Complications Between Lichtenstein Open Repair and Laparoscopic Transabdominal Pre-peritoneal (TAPP) Repair for Unilateral Inguinal Hernia

**DOI:** 10.7759/cureus.17863

**Published:** 2021-09-10

**Authors:** ZakaUllah Jan, Sajid Ali, Nisar Ahmed, Muhammad Assam Sarwar

**Affiliations:** 1 Department of General Surgery, Khyber Teaching Hospital, Peshawar, PAK; 2 Department of General Surgery, Letterkenny University Hospital, Letterkenny, IRL

**Keywords:** lichtenstein’s repair, inguinal hernia, laparoscopic tapp repair, post-operative pain, post-operative complications

## Abstract

Introduction

Laparoscopic transabdominal pre-peritoneal (TAPP) repair is a minimally invasive technique that is becoming the procedure of choice among surgeons for inguinal hernia repair and research work is still going on comparing TAPP repair with Lichtenstein open mesh repair. The objective of our study is to compare common postoperative complications in Lichtenstein mesh repair and laparoscopic TAPP repair for unilateral inguinal hernia in our unit.

Methods

Between August 2016 and August 2018, patients with unilateral inguinal hernia and ASA grade I/II were selected in the surgical outpatient department (OPD) and prospectively randomized into two equal groups. Lichtenstein open mesh repair was done in Group-I and laparoscopic TAPP repair in Group-II. The visual analog scale (VAS) was used for the assessment of the intensity of pain.

Results

A total of 100 patients with a diagnosis of unilateral inguinal hernia were included in the study. Overall, our study showed that there was less postoperative pain in those patients who underwent TAPP repair as compared to patients with Lichtenstein mesh repair (p= <0.05). There were more postoperative complications in Group-I as compared to Group-II.

Conclusion

Laparoscopic TAPP repair for inguinal hernia is associated with less postoperative pain and other postoperative complications in addition to a shorter hospital stay as compared to Lichtenstein mesh repair. Thus, this is helping in the early return of patients to daily life activities.

## Introduction

A hernia is a protrusion of a viscus or part of a viscus through the wall of the cavity in which it normally resides. Conditions like coughing, straining, obesity, and intra-abdominal malignancy can precipitate a hernia [1]. Hernias can occur in a number of different anatomical locations, including the abdominal, femoral, umbilical, and inguinal regions. The most common type of hernia is an inguinal hernia, which accounts for about 73% of all hernia cases [2]. Many different surgical procedures are performed for repairing an inguinal hernia. One of the most common operations done is mesh repair, accounting for about 700,000 hernia repairs/year in the United States [3]. However, Lichtenstein mesh repair is associated with increased chances of hematoma formation, high incidence of urinary retention, and increased postoperative pain. Recent advances in surgical procedures have brought an immense improvement in postoperative complications. Of these, minimally invasive procedures like laparoscopic TAPP, totally extraperitoneal (TEP), and intraperitoneal onlay mesh (IPOM) repair are advocated to cause decreased pain postoperatively and lowers stay in the hospital [4-5]. The aim of this study was to compare Lichtenstein mesh repair with laparoscopic TAPP repair for postoperative pain and other complications in our patients with unilateral inguinal hernia as Wright et al. in their meta-analysis showed no significant difference between the two procedures while Stoppa et al. was of the opinion that laparoscopic hernia repair had better outcomes in terms of postoperative pain and an early return to daily life physical activities [6-8].

## Materials and methods

This randomized clinical trial was conducted in the Surgical A unit at Khyber Teaching Hospital, Peshawar, from August 1, 2016, to July 31, 2018. A total of 100 patients were randomly divided into two equal groups. The inclusion criteria were patients of 18 to 70 years of age, having a unilateral inguinal hernia, patients undergoing elective surgery, and those with American Society of Anesthesiologists (ASA) grade I/II.

The exclusion criteria were patients with a diagnosis of bilateral inguinal hernia, obstructed/strangulated hernia, irreducible hernia, patients with systemic/local infection, patients with a previous history of pelvic surgery, and those with contraindications for general anesthesia or laparoscopy.

**Figure 1:**
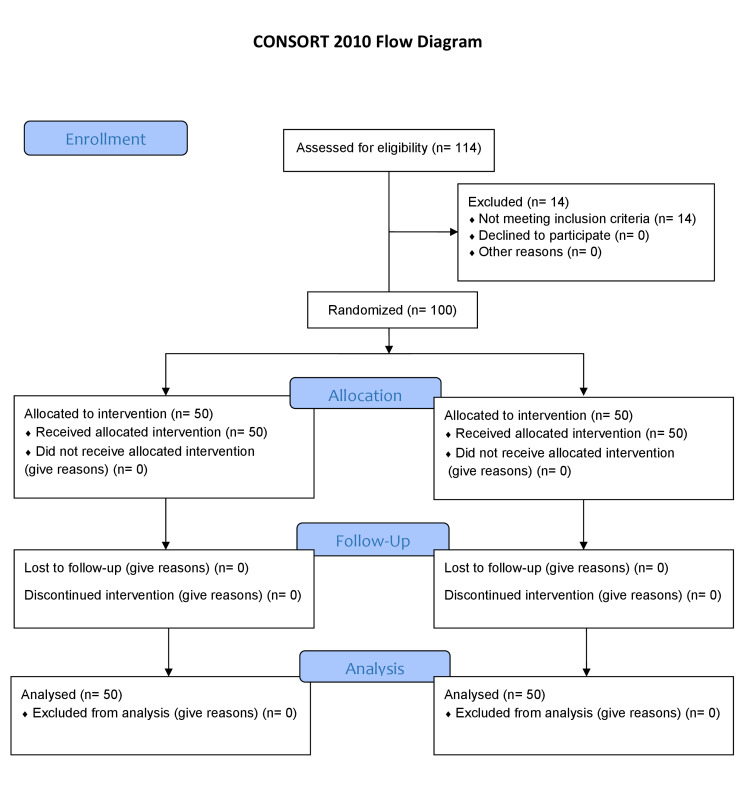
Consort diagram

After approval from the ethical committee of the hospital, patients fulfilling the inclusion criteria were randomized via simple randomization with a computer program. Informed written consent was obtained from all the patients. An anesthesia assessment was done before the procedure. All the operations were performed by the same group of surgeons having experience of more than two years in open and laparoscopic hernia surgery. All patients underwent general anesthesia. Preoperatively, investigations like full blood count (FBC), serum electrolytes, serum creatinine, serum urea, liver function tests (LFTs), viral status, random blood sugar (RBS), chest X-ray (CXR), and ECG were performed in both groups, and only patients with normal labs were included in the study. Eight patients from Group-I and six patients from Group-II were excluded due to deranged LFTs and renal functions, as shown in the consort diagram below (Figure 1).

**Figure 2:**
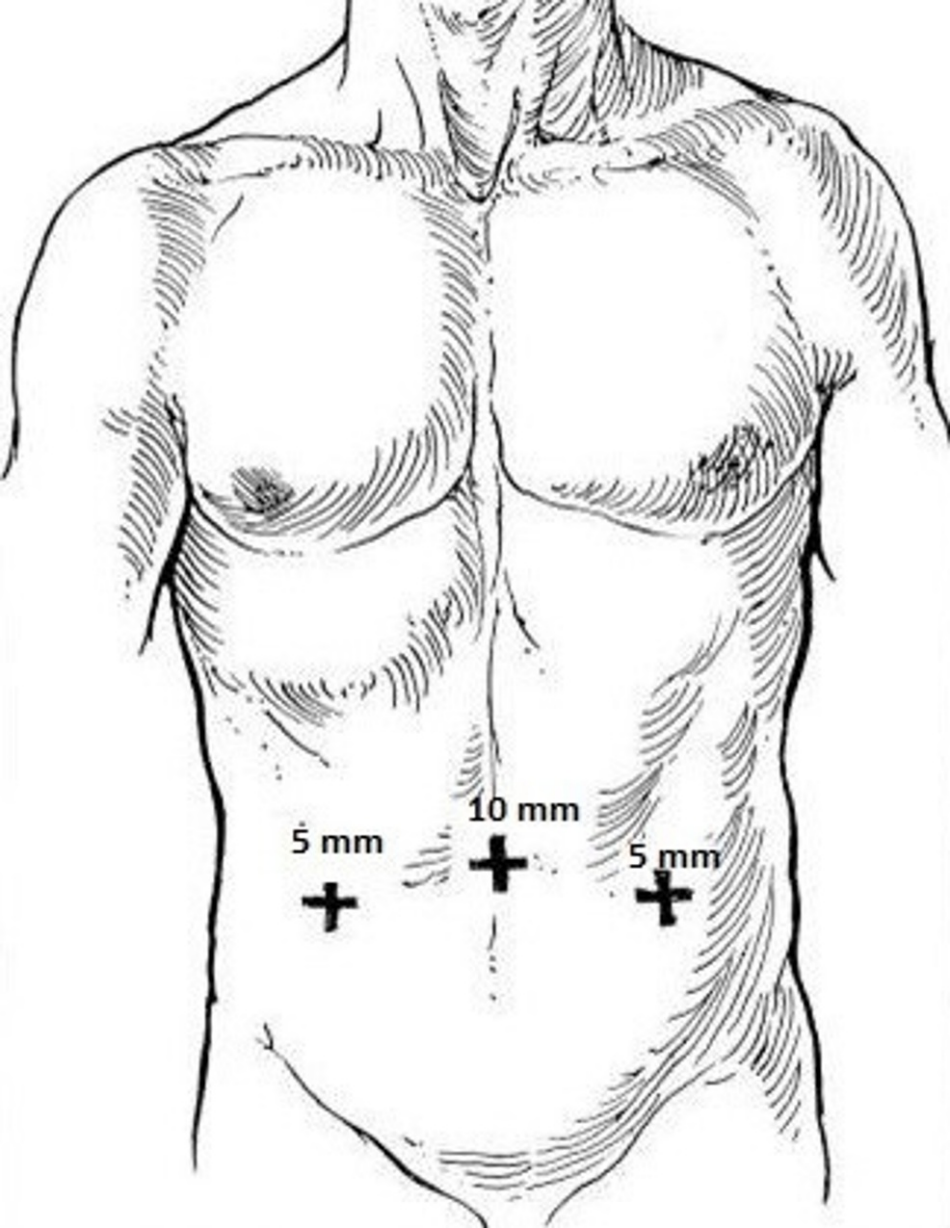
Sites for the placement of laparoscopic ports

Standard Lichtenstein mesh repair was done in Group-I patients, whereas laparoscopic TAPP repair was done in the other group according to the standard three ports technique, by first placing the umbilical (10 mm) port and then the other ports (5 mm) at the lateral border of the rectus muscle on either side of the umbilicus (Figure 2).

The visual analog scale (VAS) was used for the assessment of perception of pain, on a 0 to 10 score with 0 meaning ‘no pain’ and 10 indicating ‘worst pain’ ever experienced by a patient. VAS was performed at six and 12 hours after the operation for both groups. All the patients received the same postoperative analgesia, which includes intravenous (IV) ketorolac (30 mg) and paracetamol (1 gm) eight hourly up to 24 hours after surgery and then oral paracetamol (500 mg) 12 hourly. All patients were followed up in the clinic one week after surgery and then after three, six, and 12 months.

Data were analyzed using SPSS version 20 (IBM Corp., Armonk, NY). Quantitative variables like age and postoperative pain were calculated as mean and S.D. For qualitative data like gender, common complications like postoperative pain, hematoma/seroma formation, scrotal edema, urinary retention, and hernia recurrence were calculated as frequencies and percentages were calculated. The complications like postoperative pain, hematoma/seroma formation, scrotal edema, urinary retention, and hernia recurrence were stratified to control effect modification. Post-stratification was used through the chi-square test, keeping a <0.05 level of significance to compare the difference between the two groups.

## Results

**Figure 3:**
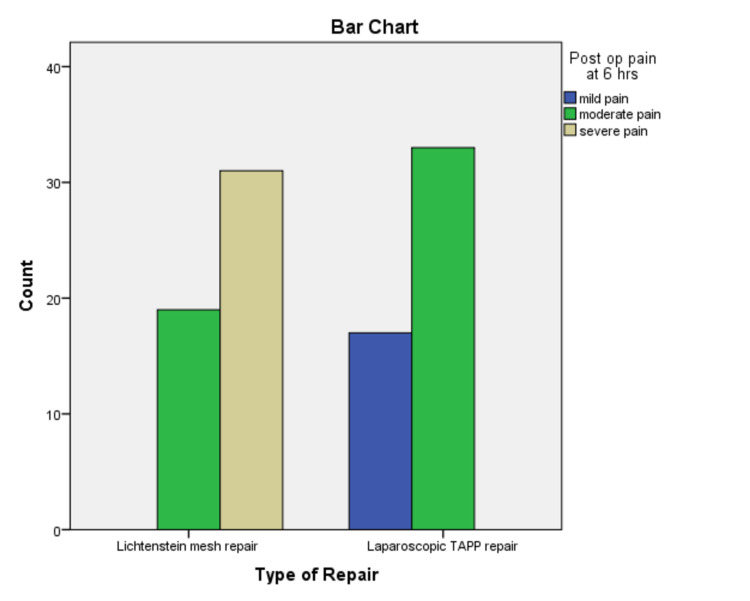
Pain at six hours postoperatively

**Figure 4:**
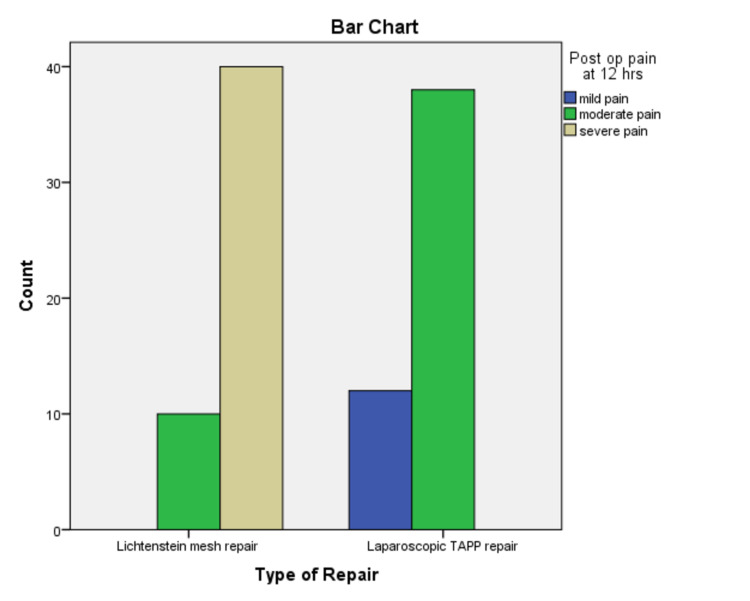
Pain at 12 hours postoperatively

The mean age was 39.40+/-16.184 years, ranging from 18 to 70 years. The mean body mass index (BMI) was 25.61+/-3.803 kg/m^2^. The baseline characteristics of the patients are shown in Table 1. There was a significant difference in pain, six and 12 hours postoperatively between the groups. At six hours postoperatively, 38% (n=19) of the patients reported moderate pain and 62% (n=31) experienced severe pain in Group-I. Whereas in Group-II, 34% (n=17) and 66% (n=33) of the patients reported mild and moderate pain respectively (p=<0.05). In addition, mild pain was experienced by 24% (n=12) of the patients and moderate pain was experienced by 76% (n=38) of the patients 12 hours after surgery in Group-II. While 20% (n=10) and 80% (n=40) of the patients reported moderate to severe pain in Group-I, respectively (p=<0.05) (Figures 3-4). The postoperative complications are shown in Table 2 and occurred in 16 patients in Group-I and three patients in Group-II. The mean length of hospital stay (LOS) was 2.15 days +/-0.557 S.D.

**Table 1:** Characteristics of patients

Characteristics	TAPP	Lichtenstein		Total Mean
Mean age (years)	40.02+/-16.226	38.78+/-16.282		39.40+/-16.184
Mean body mass index (kg/m^2^)	26.10 +/- 3.671	25.12 +/- 3.905		25.61+/-3.803
Number of patients (%); Total 100 (100)				
Male	50 (100)	50 (100)	100 (100)	
Length of hospital stay (LOS) in days	1.84 +/-0.370	2.46+/-0.542	2.15 days +/- 0.557 S.D.	

**Table 2:** Postoperative complications

Postoperative complications	Lichtenstein (n)	TAPP (n)
Hematoma formation	1	0
Seroma formation	2	0
Scrotal edema	2	0
Urinary retention	11	2
Hernia recurrence	1	1
Chronic pain	5	1

## Discussion

The principle of inguinal hernia repair is to reduce the contents, ligate the sac, and strengthen the posterior abdominal wall by placing a mesh. Traditionally, open inguinal hernia repair was a commonly performed procedure but increased postoperative pain and a delayed return to daily life activities led to the advent of minimally invasive procedures. Of these, laparoscopic TAPP repair was thought to be a safer approach.

The first laparoscopic repair for inguinal hernia was done in the 1990s [5,9]. Although laparoscopic hernia repair requires more operating time and depends on the expertise of the surgeon, it has the advantages of reduced postoperative pain, fewer postoperative complications, reduced hospital stay, and a short period of disability [10-13]. The reduced postoperative pain associated with TAPP repair is due to the fact that there is a decreased rate of postoperative complications with this procedure since postoperative pain and postoperative complications are closely related [14-17]. Due to conflicting evidence in the previous studies, we conducted a study in our tertiary care hospital to compare postoperative pain and other complications in patients undergoing both Lichtenstein repair and TAPP repair for inguinal hernia. This will help us have our local data available since local data is lacking on the given topic, and thus add to the literature.

A total of 114 patients having unilateral inguinal hernia were initially included in the study. To minimize selection bias, demographic characteristics like name, age, sex, address, phone numbers, and baseline characteristics of all patients were recorded using a standard questionnaire. The baseline characteristics in both groups were quite comparable, with two hypertensive and one diabetic patient in Group-I, and one hypertensive and two diabetic patients in Group-II. None of the patients had any bleeding disorder or prior history of ischemic heart disease. The mean age of patients in Group-I was 38.78+/-16.282 SD while that of patients in Group-II was 40.02+/-16.226 SD. All patients included in the study were males. There was zero conversion rate from TAPP to open repair.

There were more postoperative complications (32%) in Group-I compared to Group-II (4%). These included hematoma/seroma formation, scrotal edema, and urinary retention. These complications (seroma, hematoma, scrotal edema) are related to inguinal incision. Thus, they are more likely to occur in the open approach than in the laparoscopic approach [17-18]. Recurrence rates after laparoscopic inguinal hernia repair have been reported from 0% to 4% [14]. In our study, the recurrence rate was 2% and was similar between both groups.

Chronic postoperative pain is a problem after open repair. Various studies suggest that pain at one year has been reported by up to 30% of patients following surgery for inguinal hernia [19]. In our study, chronic pain was significantly higher in Group-I (TAPP: 2%; Lichtenstein repair: 10%) as also mentioned in other studies [12,20-21]. The main reason may be the different space placement of mesh compared with the open approach. However, this needs further study. Hematoma and seroma formation are also considered risk factors for chronic pain following inguinal hernia repair [17,22-23].

As these are higher in Group-I, thus they justify the higher incidence of chronic pain in Group-I.

The results of our study also confirm that LOS was significantly lower in Group- II as compared with Group-I. The increased LOS in Group-I (2.46 vs 1.84) might be due to the high postoperative pain and complication rate.

## Conclusions

Our study revealed that with laparoscopic TAPP repair for unilateral inguinal hernia, there is less postoperative pain. In addition, there are fewer postoperative complications and a shorter hospital stay as compared to open Lichtenstein mesh repair. Hence, the trend is changing to laparoscopic TAPP repair, thus aiding in an early return of the patient to normal physical activities.
